# Rennet-Induced Casein Micelle Aggregation Models: A Review

**DOI:** 10.3390/foods11091243

**Published:** 2022-04-26

**Authors:** Daniel Salvador, Yoseli Acosta, Anna Zamora, Manuel Castillo

**Affiliations:** 1 Department of Agroindustrial Science, National University of Trujillo, AV. Juan Pablo II s/n—University City, Trujillo 13011, Peru; dsalvador@unitru.edu.pe; 2 School of Agroindustrial Engineering, National University of Trujillo, AV. Juan Pablo II s/n—University City, Trujillo 13011, Peru; yacosta@unitru.edu.pe; 3 Department of Animal and Food Science, Centre d’Innovació, Recerca i Transferència en Tecnologia dels Aliments (CIRTTA), Universitat Autònoma de Barcelona, Travessera dels Turons s/n, Bellaterra, 08193 Barcelona, Spain; anna.zamora@uab.cat

**Keywords:** enzymatic coagulation, hydrolysis, κ-casein, clotting time, gelation time, coagulation time, micellar aggregation, gel assembly, gel firming, gel hardening

## Abstract

Two phases are generally recognized in the enzymatic coagulation of milk: hydrolysis and aggregation, although nowadays more and more researchers consider the non-enzymatic phase to actually be a stage of gel formation made up of two sub-stages: micellar aggregation and hardening of the three-dimensional network of para-κ-casein. To evaluate this controversy, the main descriptive models have been reviewed. Most of them can only model micellar aggregation, without modeling the hardening stage. Some are not generalizable enough. However, more recent models have been proposed, applicable to a wide range of conditions, which could differentiate both substages. Manufacturing quality enzymatic cheeses in a cost-effective and consistent manner requires effective control of coagulation, which implies studying the non-enzymatic sub-stages of coagulation separately, as numerous studies require specific measurement methods for each of them. Some authors have recently reviewed the micellar aggregation models, but without differentiating it from hardening. Therefore, a review of the proposed models is necessary, as coagulation cannot be controlled without knowing its mechanisms and the stages that constitute it.

## 1. Introduction

**Figure 1:**
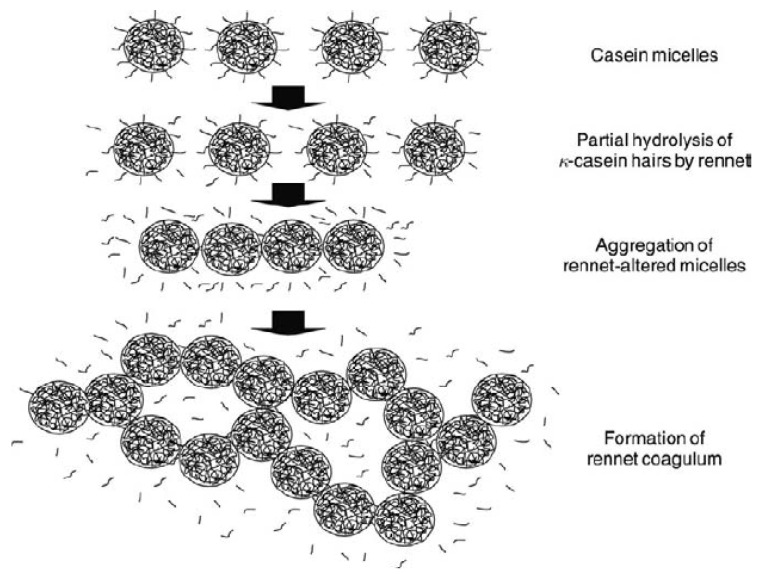
Milk coagulation process. Reproduced with permission from [Lucey, J.], [Rennet-Induced Coagulation of Milk. In *Encyclo-pedia of Dairy Sciences*]; published by [Elsevier], [2011].

The coagulation or gelation of milk after the addition of a proteolytic enzyme (e.g., chymosin) is usually divided into two overlapping phases: the enzymatic phase and the non-enzymatic or aggregation phase [1]. This division into two stages has been used by various authors [2,3,4,5]. In the first stage, the hydrolysis of κ-casein, located on the micellar surface, destabilizes the micelles both electrostatically and sterically, triggering, in the presence of Ca^2+^, the micellar aggregation process [6]. Hardening is typically considered an extension of aggregation, but, indeed, it seems not to be the case, as the study of this step actually requires a different approach and instrumentation compared to aggregation. In fact, gel hardening is the most complex and currently least understood aspect of enzymatic milk coagulation. Various instruments are available to study gel rheological properties that are critical for cheese manufacturing control, such as the rheologically determined gelation time or the appropriate elastic modulus for cutting [7]. As a result, there is particular interest in developing methods that can be used to study/quantify gelation in the cheese vat [8], as control of the gel hardness is critical for the objective selection of the optimal curd cutting time that affects both cheese quality and yield. Figure 1 outlines the coagulation process of milk.

**Figure 2:**
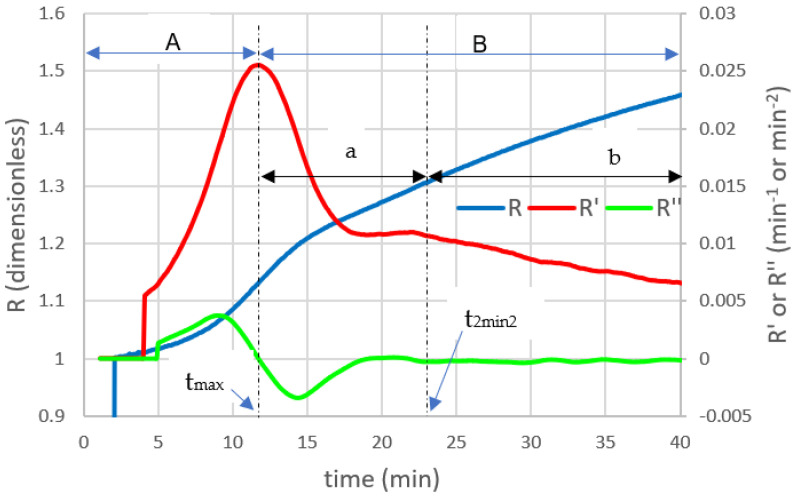
Coagulation curves of pasteurized whole milk. R is the light scattering ratio, R’ and R″ are the first and second derivatives of R, respectively. t_max_ is the time to the maximum of the first derivative of R. t_2min2_ is the time to the second minimum of the second derivative of R. A is the enzymatic period. B is the nonenzymatic period. a is the aggregation period. b is the hardening period. Reproduced with permission from [Payne, F.A.; Castillo, M.], [Light Backscatter Sensor Applications in Milk Coagulation. In *Encyclopedia of Agricultural, Food, and Biological Engineering*]; published by [Taylor & Francis], [2007].

Payne and Castillo [9], who studied milk coagulation using an NIR light scattering sensor, described in the sigmoidal curve generated by the instrument response two periods that overlap during coagulation (Figure 2): (A) the period corresponding to the phase of enzymatic hydrolysis of micellar destabilization, which goes from the addition of the enzyme to approximately the inflection point of the light scattering ratio (R) or time corresponding to the maximum of the first derivative of R (t_max_); and (B) the period corresponding to the non-enzymatic phase of coagulation which consists of two phases: (a) the period corresponding to the aggregation phase of the destabilized micelles that goes from t_max_ until the moment in which the gel begins to offer mechanical resistance (e.g., the point that could be identified, approximately, as the time at which a value of G′ = 1 Pa is reached, determined by a rheometer [10,11], related to t_2min2_ [12]) and (b) the asymptotic period corresponding to the hardening phase of the casein gel that continues until the optimum curd cutting point is reached. Harboe et al. [13] and Fox et al. [8] also describe differences between the aggregation phase and the hardening phase, but from the point of view of changes in viscosity and rheological parameters. Horne and Lucey [14] carried out the last review of the aggregation models but, as mentioned above, without differentiating the aggregation phase from the hardening phase. As seen above, the controversy raised by the separation of the non-enzymatic phase of milk coagulation into phases of aggregation and hardening will serve as the basis for studying, in a first approach, the different models that describe the phenomenon of micellar aggregation.

## 2. Micellar Aggregation

**Figure 3:**
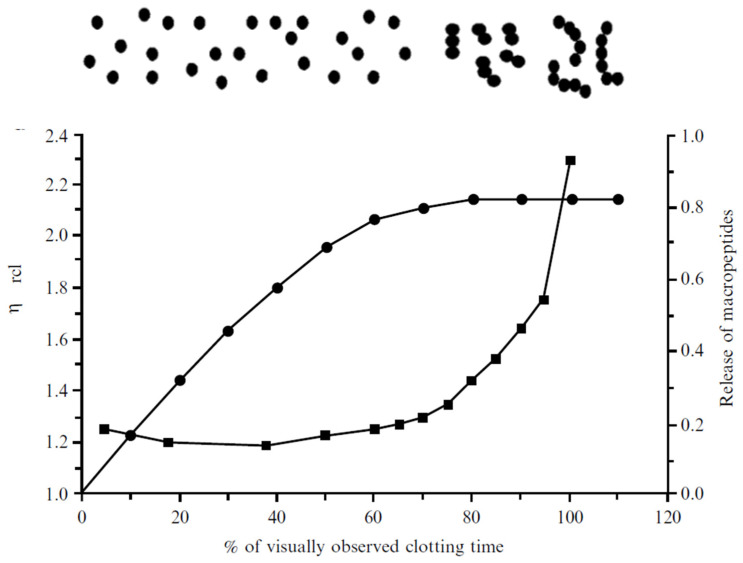
Release of macropeptides, expressed as fraction (●). changes in relative viscosity (■) during the course of rennet coagulation. On top of the figure, casein micelle aggregation progress is graphically represented. Reproduced with permission from [Fox, P.F.; Guinee, T.P.; Cogan, T.M.; McSweeney, P.L.H], [*Fundamentals of Cheese Science*, 2nd ed.]; published by [Springer], [2017].

After the enzymatic hydrolysis of κ-casein, the curd formation phase begins, consisting, in the first instance, in the aggregation of hydrolyzed casein micelles. Aggregation of destabilized micelle begins when there is sufficient hydrolyzed κ-casein 65–90%, according to [15], and if the activity of calcium ions and temperature are adequate [16]. Initially, small aggregates are formed in the form of chains (Figure 3), instead of lumps [6]. As more micelles are aggregating, the apparent viscosity and the amount of casein macropeptide (CMP) released are observed to increase. Chain formation could be explained using the geometric model described by Dalgleish and Holt [17], who state that two hydrolyzed casein micelles will only aggregate if they have sufficiently large holes on their surface formed by hydrolyzed κ-casein and if they collide at the proper orientation. The inter-particle bonds could be established through calcium bridges and/or hydrophobic interactions [8]. Modification of histidyl, lysyl, and arginyl residues in κ-casein inhibits the aggregation phase. This finding suggests that a positively charged group on para-κ-casein reacts electrostatically with unidentified negative charged sites. In native micelles, this positive site may be shielded by the CMP segment of κ-casein, becoming exposed and, subsequently, reactive when this peptide is released [18]. Changes in the surface hydrophobicity of casein micelles during the gelation process have also been confirmed using a fluorescent marker, 8-anilino naphthalene-1-sulfonate [19,20].

The aggregation is due in part to the van der Waals attraction, but it also requires participation of Ca^2+^, as the van der Waals attraction is not sufficient. The need of a sufficient amount of Ca^2+^ in the serum phase of milk clearly shows its role. Presumably, the effect of Ca^2+^ ions is double. First, Ca^2+^ decreases electrostatic repulsion by neutralizing negative charges on the micelles [21], being more efficient than H^+^ ions in the pH range under consideration. Second, Ca^2+^ ions can bridge between negative sites on paracasein micelles by means of salt bridges. It is very well-known that lowering the pH of milk considerably increases Ca^2+^ activity [22] by shifting the existing ionic balance between the soluble and micellar phases, releasing H^+^ from the micelle.

**Figure 4:**
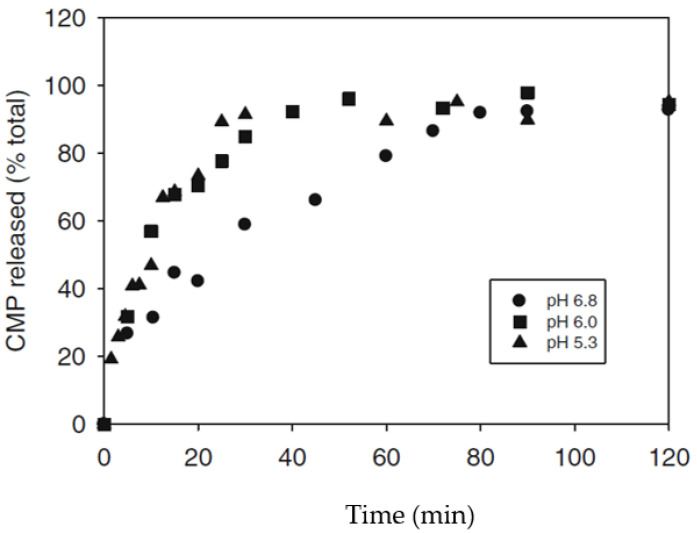
Release of casein macropeptide during coagulation of milk at different pH values. Reproduced with permission from [Corredig, M.; Salvatore, E.], [Enzymatic Coagulation of Milk. Advanced Dairy Chemistry: Volume 1B: Proteins: Applied Aspects]; published by [Springer], [2016].

Figure 4 shows the percentage of CMP released as a function of time at different pH values. It can be observed that, as the pH decreases, less time is needed to release the amount of CMP necessary to cause the aggregation of the micelles [23], as acidification progressively solubilizes the micellar calcium phosphate, releasing ionic calcium, bringing forward the start of the aggregation phase [24]. The Berridge clotting time or visual coagulation time is the time at which the first flocs of hydrolyzed casein are visible on the walls of a glass tube [25,26]. The primary enzyme reaction is ~85% complete at a time equivalent to 60% of the visual clotting time. Between 60 and 80% of the visual coagulation time, the rennet-destabilized micelles start to aggregate gradually [8].

Castillo et al. [27], coagulating goat milk, found that the start of the aggregation phase could be objectively determined using a NIR light scattering sensor, by the inflection point of the scattering ratio, R, (Figure 2), which corresponded to 88% of the Berridge coagulation time. Tabayehnejad et al. [28], working with low heat skimmed milk powder and with the same methodology, found that the start of aggregation occurs at 84% of the Berridge coagulation time, confirming two undeniable facts: that the detection of an aggregation point mainly depends on the sensitivity of the measurement technique to the presence of the aggregates and also that the two processes (proteolysis and subsequent aggregation) overlap in time [4]. Therefore, the value of t_max_ (Figure 2) was proposed as an approximate target value of aggregation start time [28,29].

## 3. Aggregation Models of Hydrolyzed Casein Micelles

Numerous efforts have been made to mathematically describe the aggregation reaction of hydrolyzed casein micelles. They have been proposed from simpler models, fundamentally empirical, to more sophisticated ones, based on computational simulations of Brownian dynamics, which constitute clear evidence of the interest in knowing the behavior of this phase of coagulation. Next, the main models that have been studied/proposed will be described.

### 3.1. Holter Model

Almost 150 years ago, Storch and Segelcke (1874) (cited by McMahon et al. [30]) found that the coagulation time is inversely proportional to the enzyme concentration, according to the formula:

(1) $$t_{c}E=k$$

where t_c_ is the coagulation time, E is the enzyme concentration, and k is the constant.

It was soon found that this relationship only holds in a small range of pH, temperature, and enzyme concentration. For this reason, Holter [31] separately considered the duration of the hydrolysis phase (t_h_) and the aggregation time (t_a_), defining the coagulation time as t_c_ = t_h_ + t_a_. Subsequently, Foltmann [32], assuming Holter’s proposal, proposed the following equation for t_c_:

(2) $$t_{c}=\frac{C}{\left[E\right]}+t_{a}$$

where t_a_ is the aggregation time, t_c_ is the coagulation time, [E] is the enzyme concentration, and C is the constant.

**Figure 5:**
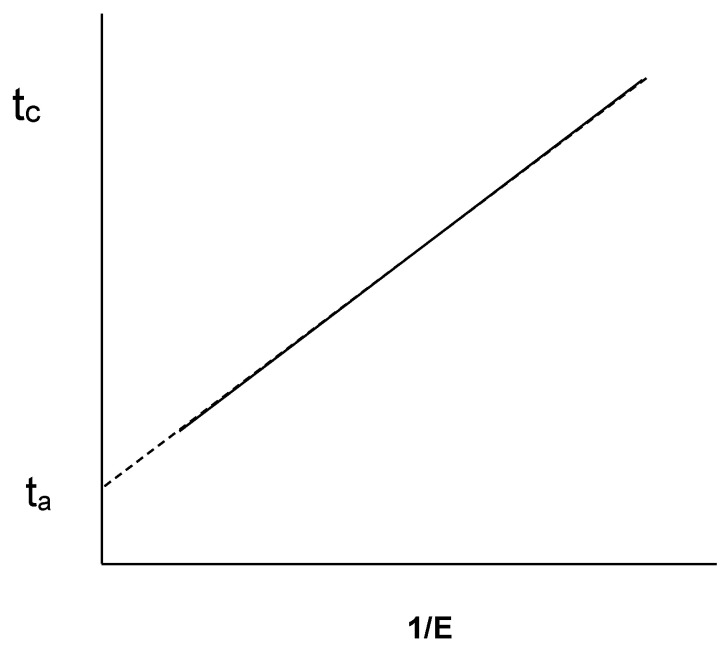
Relationship between enzyme concentration (E), aggregation time (t_a_), and coagulation time (t_c_). Reproduced with permission from [Dalgleish, D.G.], [A New Calculation of the Kinetics of the Renneting Reaction.]; published by [*J. Dairy Res.*], [1988] [34].

In this model, the intercept, t_a_, would come to represent the minimum duration of coagulation when the enzyme concentration tends to infinity (i.e., hydrolysis would be instantaneous, not limiting the aggregation rate). As can be seen, this relationship is purely empirical, but it is a relevant relationship that must be satisfied as a starting point for any more descriptive mechanistic models. Figure 5 shows the representation of Equation (2). The equation assumes that there is no overlap between proteolysis and the aggregation phase, and that the degree of proteolysis is always the same at the time of coagulation [33].

### 3.2. Payens Model

Payens [35], Payens et al. [36], and Payens and Wiersma [37] proposed a model for aggregation based on Michaelis–Menten kinetics for hydrolysis and Smoluchowski aggregation kinetics. They considered that the extent of micelle aggregation is proportional to the amount of cleaved κ-casein. Thus, in the early stages of the reaction, small concentrations of micelles that can aggregate may have been originated by hydrolysis [38]. Therefore, there will be a lag period during which no great effect of the enzyme on the average particle size will be observed, proposing the following model for the coagulation time:

(3) $$t_{c}\approx \sqrt{\frac{2}{k_{s}V_{\max}}}$$

where t_c_ is the coagulation time, k_s_ is the aggregation rate constant, and V_max_ is the maximum rate of hydrolysis of κ-casein.

This model described well the coagulation kinetics of isolated κ-casein generated with pepsin [39]. The main problem with the model was that the rate constant for aggregation (k_s_) of the hydrolyzed micelles was very low compared to the constants found experimentally. This has been attributed to the fact that k_s_ is an average of all values, from very low values at the beginning and high values at the end of the aggregation reaction [3,40,41,42].

### 3.3. Step Function Model

Dalgleish [43] proposed the step function model using a binomial distribution. This model is based on the idea that the enzyme would act on the surface of the micelle in a random way and that a critical level of proteolysis must be reached before aggregation is possible. The model assumes that the hydrolysis and aggregation phases can be modeled taking into account that ~97% of the κ-casein in a micelle needs to be hydrolyzed for it to participate in aggregation.

Applying a binomial distribution to the hydrolysis phase controlled by Michaelis–Menten kinetics and Smoluchowski aggregation kinetics, we obtain:

(4) $$t_{c}=t_{h}+t_{a}\;\sim \;\frac{k_{M}}{V_{\max}}\ln\left[\frac{1}{\left(1-\alpha_{c}\right)}\right]+\frac{\alpha_{c}.{\;S}_{0}}{V_{\max}}+\frac{1}{2k_{s}.{\;C}_{0}}\left[\frac{M_{\mathrm{crit}}}{M_{0}}-1\right]$$

where t_c_ is the coagulation time, t_h_ is the hydrolysis time, t_a_ is the aggregation time, k_M_ is the Michaelis–Menten constant (it approximates the dissociation constant for the enzyme-substrate complex), V_max_ is the maximum rate of proteolysis at infinite substrate concentration, α_c_ is the extent of κ-casein hydrolysis, S_0_ is the initial concentration of κ-casein, k_s_: is the aggregation rate constant, C_0_ is the concentration of aggregating material, M_crit_ is the average molecular weight at coagulation time (≈10 micellar units), and M_0_ is the average molecular weight at t = 0.

This calculation method for chymosin-induced casein micelles aggregation is similar to that of Payens [36], but it incorporates certain important differences such as the use of full Michaelis–Menten kinetics and the description of the aggregation mechanisms based on probability functions. The calculations agree well with the experimental evidence, in terms of enzyme variation and substrate concentration [44]. If there is any inconvenience in Dalgleish’s model, it is its complexity, as an analytical solution is not reached, and simplifications are needed to obtain such solutions [3].

### 3.4. The Energy Barrier Model

The energy barrier model proposed by Darling and van Hooydonk [45] is based on the idea that an energy barrier (Equation (5)) is gradually lowered due to proteolysis, leading to an increasing probability of collision during the reaction of aggregation. The stability factor, W, characterizes the relationship between the particle collision frequency and the frequency with which these collisions are successful, resulting in aggregation. The coagulation of casein micelles by rennet action can then be satisfactorily described by determining the relationship between the stability factor W, which represents a measure of the energy barrier, and the concentration of κ-casein as a function of time [45].

(5) $$W_{t}=W_{0}\exp\left(-C_{m}.V.t\right)$$

where W_t_ is the stability factor at time t for casein micelles, W_0_ is the initial stability factor for casein micelles, C_m_ is the constant that relates the stability of the casein micelle to the concentration of κ-casein, V is the enzymatic reaction rate, and t is the time.

Considering first-order enzyme kinetics (i.e., simplification from Michaelis–Menten kinetics) for the hydrolysis phase and Smoluchowski aggregation kinetics, Equation (6) is obtained for the coagulation time, t_c_:

(6) $$t_{c}=\frac{1}{V}\left[S_{0}+\frac{1}{C_{m}}\left(\exp\left(-C_{m}.S_{0}\right)-1\right)\right]+\frac{W_{0}\exp\left(-C_{m}.S_{0}\right)}{k_{s}}\left[\frac{1}{n_{c}}-\frac{1}{n_{0}}\right]$$

where t_c_ is the coagulation time, S_0_ is the initial concentration of κ-casein, k_s_ is the aggregation rate constant, n_c_ is the concentration of casein aggregates at time t_c_, and n_0_ is the initial concentration of casein micelles.

The model describes the variations observed in the coagulation time (t_c_) as a function of temperature, rennet concentration, and total protein concentration. Additionally, it demonstrated the existence of a delay time in the start of aggregation equivalent to 60% clotting time [45]. This energy barrier model has been tested extensively [4]. The difficulty with the model lies in its simplification of Michaelis kinetics to allow calculations [39].

### 3.5. The Functionality Model

Hyslop [46] and Hyslop and Qvist [47] proposed the functionality model, which is based on the fact that the continuous removal of CMP would lead to multiple holes on the surface of the micelle and that, therefore, the aggregation rate constant, k_ij,_ should be a function of the number of free reactive sites on the aggregation particles, which met the required conditions for a polyfunctional model of the Flory–Stockmayer type [48]:

(7) $$k_{\mathrm{ij}}=K\left[4+2\left(f-2)(i+j\right)+{\left(f-2\right)}^{2}\mathrm{ij}\right]$$

where K is the proportionality factor, f is the number of functional sites (functionality), and I and j are the number of aggregating particles of type i and j, respectively.

If f = 1, only dimer formation is possible, while if f = 2, the model predicts linear polymers and if f > 2, chain branching occurs and gelation is possible. Initially, at time t = 0, f = 0, the micelles have no reactivity or inclination to aggregate [4]. Although the Stockmayer expression (Equation (7)) does not include explicit geometric assumptions, it is generally assumed that the aggregating entities are large extended polymers in which many functional sites are possible, and in which steric problems are improbable. With spherical particles, such as casein micelles, steric problems could easily appear when two micelles become close enough. Therefore, K, f, or both in Equation (7) would be expected to be low. However, Stockmayer’s expression would still apply [3].

### 3.6. Adhesive Hard-Sphere Model

**Figure 6:**
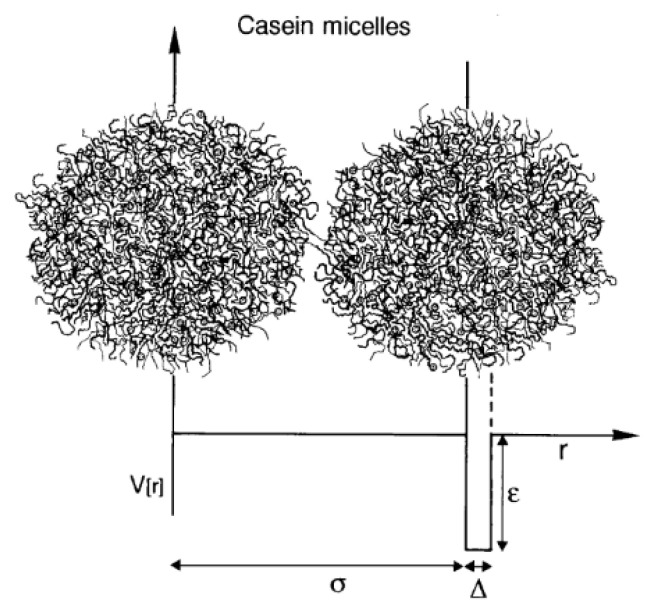
Two interacting casein micelles. The hard-core repulsion is preceded by an attractive interaction modeled as a “square well” potential. The depth of the well depends on the stability of the κ-casein brush. Reproduced with permission from [De Kruif, C.G.], [Casein Micelle Interactions]; published by [*Int. Dairy J.*], [1999].

Reactions leading to aggregation of hydrolyzed micelles have also been studied using the adhesive hard-sphere theory, proposed by de Kruif [49,50]. Instead of attributing the increase in viscosity to the formation of aggregates, de Kruif proposed a different approach based on considering casein micelles as rigid, sterically stabilized spheres that become sticky, or adhesive, as the κ-casein is being proteolyzed and released. The relative viscosity, n_r_, of skim milk is described as:

(8) $$n_{r}=1+2.5\Phi +\left(5.9+\frac{1.9}{\tau }\right)\Phi^{2}$$

where Φ is the particle volume fraction, which varies as hydrolysis and aggregation occurs, and τ is a stickiness parameter related to the depth, ε, of an attractive potential created when κ-casein pili (i.e., CMP) are separated from the casein micelle surface by rennet. Figure 6 shows two interacting casein micelles, where V(r) is the potential of the “square well”, with width Δ and depth ε, and σ is the diameter of the hard core.

**Figure 7:**
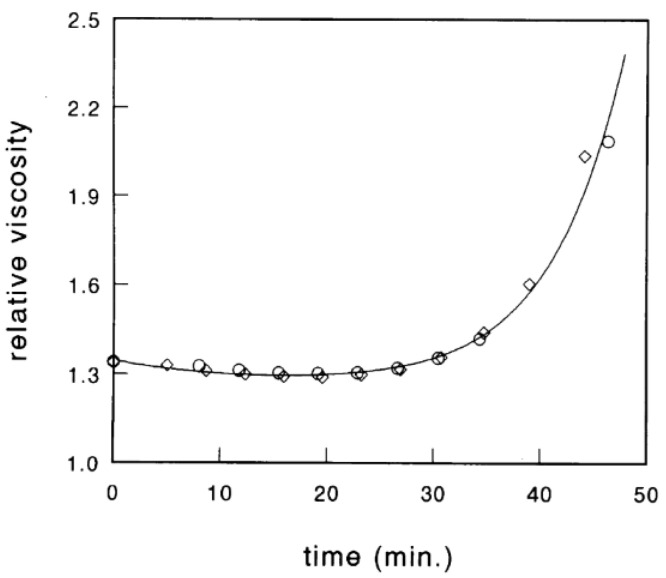
Relative viscosity of skim milk during the coagulation process. The drawn line represents the theoretical calculation in which the micelles are considered to be sticky hard spheres. Reproduced with permission from [De Kruif, C.G.], [Casein Micelle Interactions]; published by [*Int. Dairy J.*], [1999].

Chymosin induces an initial decrease in viscosity that exceeds a minimum before an exponential increase takes places as coagulation progresses (Figure 7). Therefore, rotational viscometers provide limited information about coagulation, as during this process, the viscosity of milk tends towards infinity at visual coagulation time [51].

The initial decrease in viscosity arises due to the decrease in the particle volume fraction, Φ, when the hairy layer of κ-casein is hydrolyzed. This decrease in hydrodynamic size has been measured experimentally using dynamic light scattering techniques in diluted [52] and concentrated [53] micellar suspensions. Subsequently, the viscosity starts to increase due to aggregation and network formation. An important disadvantage of the hard-sphere model is that the time dependence of relative viscosity is mostly related to the proteolysis reaction, which limits its use to preliminary stages of aggregation [54]. Indeed, according to published data, the hard-sphere model only seems to describe the coagulation process up to the visual coagulation time, due to the existence of an asymptote at about 100% visual clotting time, ~50 min in Figure 7. As it can be observed in this figure, theory has successfully estimated viscosity changes up to the point where aggregation occurs [5]. As a result, it cannot provide information on the complete kinetics of aggregation and gel firming, two highly significant events in the definition of the gel properties [4].

### 3.7. Fractal Aggregation Model

Bremer et al. [55,56,57] applied fractal theory to acid-induced casein particle aggregation. It has also been demonstrated that rennet-induced aggregation of casein micelles can be modeled according to fractal mechanisms [58,59,60,61,62]. Aggregates can be considered fractals if their geometric structure is similar when viewed over a reasonably large range of scales (that is, irrespectively of the observation scale selected). As the number of particles in an aggregate increase with time, a relationship between the size of an aggregate and the number of particles, N_p_, is obtained as follows:

(9) $$N_{p}={\left(\frac{R}{a}\right)}^{D}$$

where R is the radius of the aggregate, a is the radius of the primary particle, and D is the fractal dimension.

Assuming a sphere of radius R containing a number, Ns, of closely packed particles, the volumetric fraction of the particles in a fractal aggregate, φ_A_, is given by:

(10) $$\varphi_{A}=\frac{N_{p}}{N_{s}}={\left(\frac{R}{a}\right)}^{D-3}$$

As D < 3 invariably (in three-dimensional space), this means that the particle volume fraction φ_A_ will decrease with increasing values of R as the group grows [63]. At a certain radius (R_g_), to which the aggregate is known as the fractal ‘blob’, φ_A_ will be equal to the volume fraction of particles in the system φ and all aggregates will touch, forming a continuous gel network [5]. Bremer et al. [55] defined the gelation point base on this event, which infers that all the particles present in the system are incorporated into the aggregates. The real question is whether this corresponds to the experimentally measured rheological gel point (crossover point—when the elastic modulus, G′, becomes higher that the viscous modulus, G″, which is sometimes generalized empirically as G′ = 1 Pa); this seems to be the assumption made [4]. Furthermore, assuming that the fractal blob coincides with the moment in which the rheometer yields a measurable rheological response, the obvious question is how the increase in the elastic modulus of the structure takes places, according to the fractal model [64].

**Figure 8:**
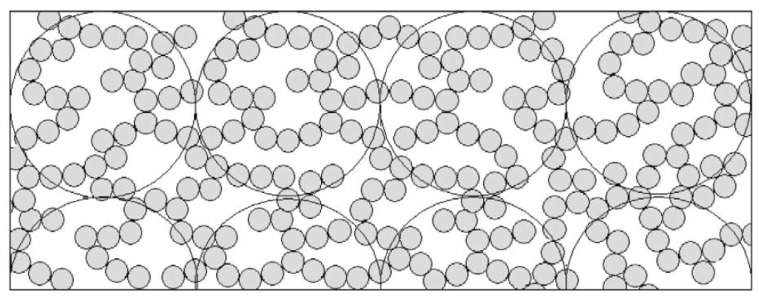
Formation of a weak gel in which most of the particles are weakly bound to the network. Larger circles represent the fractal ‘blob’ concept. Reproduced with permission from [Horne, D.S.], [Banks, J. Rennet-induced Coagulation of Milk. In *Cheese Chemistry, Physics and Microbiology*]; published by [Elsevier], [2004].

According to [4], the fractal network structure is similar to the image represented in Figure 8.

**Figure 9:**
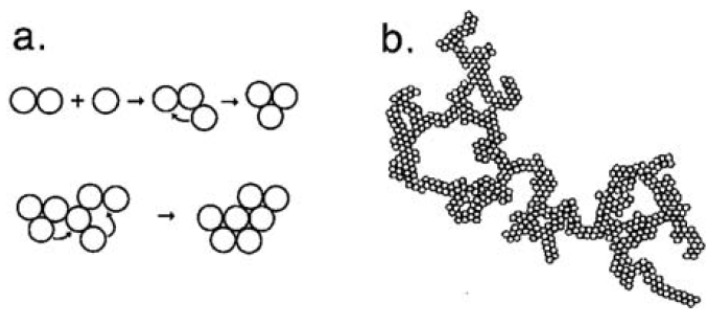
Short-term rearrangement. (**a**) Particle rearrangement. (**b**) Fractal group in two dimensions, where a short-term rearrangement has occurred. Reproduced with permission from [Walstra, P.], [*Physical Chemistry of Foods*]; published by [Marcel Dekker], [2003].

Walstra [63] mentioned that an intermicellar rearrangement is also possible as the aggregates are formed (short-term rearrangement); the particles wrap around each other until they have obtained a higher coordination number, which indicates a more stable configuration (Figure 9).

The fractal dimension of casein gels obtained by enzymatic coagulation can be obtained using techniques such as permeametry, turbidimetry, or rheology, reporting values for this type of gel from 2.17 to 2.4 [44].

The fractal model can describe the aggregation of casein micelles until the formation of the network occurs, at which point the fractal structure would slowly disappear due to a massive restructuring of the aggregates, producing greater compaction of the aggregates and creating larger pores in the gel [65,66]. It is therefore not possible to efficiently model the hardening stage of the gel formation.

The various models mentioned throughout this review essentially describe the increase in average molecular mass of the micellar aggregates as a function of time. The average molecular mass is easily measurable by static light scattering techniques [4]. However, due to the problems encountered with multiple light scattering, where the detected photon are scattered several times as they travel through the suspension media due to the presence of multiple scatterers, these techniques are applicable only to highly dilute suspensions [67] or over very short path lengths, as in the turbidity measurements [68]. A main disadvantage of all these models is that most describe the total or partial hydrolysis and aggregation of casein micelles, but in no case the complete process, largely due to limitations related to experimental techniques.

### 3.8. Light Scattering Model

A method that has apparently overcome the aforementioned drawbacks is the one proposed by Castillo et al. [29], describing the kinetics of light scattering change during the aggregation steps of hydrolyzed casein micelles and subsequent gel hardening. This model could differentiate the aggregation stage from the hardening stage. It combines second order kinetics to describe the aggregation of casein micelles and first order kinetics that would correspond to casein gel hardening.

In particular, the model considers the following second-order reaction for the aggregation stage:

(11) $$R_{a}=R_{\infty A}-\left[\frac{R_{\infty A}-R_{0A}}{1+\left(R_{\infty A}-R_{0A}\right)k_{2}\left(t^{\prime }-t_{\max}\right)}\right]$$

where R_a_ is the diffuse reflectance ratio attributed to the aggregation period; R_∞A_ is the diffuse reflectance ratio at t = ∞, during the aggregation period; R_0A_ is the diffuse reflectance ratio at t_max_; k_2_ is the second order reaction rate constant; t′ is the time after enzyme addition; and t_max_ is the starting time for the aggregation reaction.

**Figure 10:**
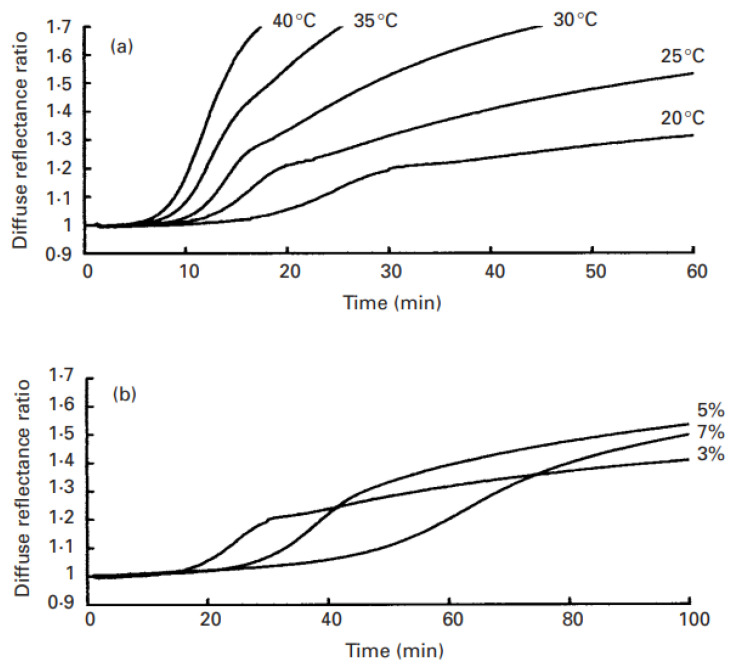
Effect of temperature and protein concentration on apparition of a shoulder in the curve of diffuse reflectance ratio versus time. (**a**) Data corresponded to skim milk adjusted to 30 g protein/kg and coagulated at 20, 25, 30, 35, and 40 °C and at constant calcium chloride and enzyme concentration. (**b**) Data corresponded to skim milk adjusted to 30, 50, and 70 g protein/kg and coagulated at 20 °C and at constant calcium chloride and enzyme concentration. Reproduced with permission from [Castillo, M.Z.; Payne, F.A.; Hicks, C.L.; Laencina, J.S.; López, M.-B.M.], [Modelling Casein Aggregation and Curd Firming in Goats’ Milk from Backscatter of Infrared Light.]; published by [*J. Dairy Res.*], [2003].

Applying light scattering to monitor milk coagulation by rennet, Castillo et al. [29] observed a systematic distortion of the scattered light profile during coagulation. At low protein concentration and temperature, a distinct shoulder separated the light scattered coagulation profile, which tended to disappear with increasing temperature and protein concentration (Figure 10). This behavior was attributed to the presence of two distinct but partially overlapping phenomena: the aggregation of casein micelles and the hardening of the three-dimensional gel structure. Apparently, these two stages of gel formation were not clearly distinguished at high protein concentration and temperature, possibly because these coagulation conditions facilitated the overlap of these mentioned phenomena.

The possible aggregation mechanisms involved in casein gels are now being studied using computational simulations of Brownian dynamics [5,69]. With this type of approach, the role of repulsive barriers and different bonds types, as well as the behavior/movements/collisions of the particle on the aggregation mechanisms and kinetics, can be studied and better understood. In other words, this technique is very useful in our understanding of how particle interactions influence gelation properties [5]. New techniques for monitoring milk coagulation are also being tested, such as photon density wave spectroscopy (PDW) [70], fast Fourier transform continuous cyclic voltammetry [71], and fluorescence [72], demonstrating the continuing interest in finding new methods to improve the control of milk coagulation.

## 4. Summary

The study and modeling of the aggregation of enzymatically hydrolyzed casein micelles is essential to have a better understanding of this stage of milk coagulation. This would allow us to achieve a better control of the production of enzymatically coagulated dairy products.

The models formulated to date for the aggregation phase have some limitations and some advantages. Holter’s model [31], modified by Foltmann [32], assumes that there is no overlap between proteolysis and the aggregation phase, and that the degree of proteolysis is always the same at the time of coagulation. In the model proposed by Payens [35,36,37], which was one of the first based on an aggregation kinetic, it was observed that the rate constant for the aggregation of hydrolyzed micelles is very low compared to the constants found experimentally. Dalgleish’s step function model [43], which was one of the first models to mention that a certain degree of hydrolysis of casein micelles needs to be reached before they can aggregate, is very complex, and simplifications have to be made to obtain analytical results. The step function model does not explain why a certain degree of hydrolysis of the casein micelles has to be achieved before they aggregate. In an attempt to solve this problem, Darling and van Hooydonk [45] proposed the energy barrier model, which has been extensively tested. However, it requires a simplification of Michaelis kinetics to allow calculations. The functionality model of Hyslop and Qvist [46,47], which hypothesized that the rate constant of aggregation depends on the number of active free sites in casein micelles, is based on the assumption that the aggregating particles are mostly linear, with many functional points. The adhesive hard sphere model of de Kruif [49,50], which describes the experimental data well until the end point of aggregation, does not model the hardening stage, so it is mostly related to the proteolysis reaction and the initial phase of micellar aggregation. Regarding the fractal aggregation model of Bremer et al. [55,56,57], it has to be assumed that the value of the fractal dimension is constant throughout the aggregation stage. However, when the gel is formed, this fractal structure tends to disappear. As a result, the model does not provide information about the next stage, the gel hardening. It has been observed in some cases that these models are formulated to model micellar aggregation up to a certain point, while others have difficulties in extrapolating their results to other, more concentrated systems. However, light scattering model proposed by Castillo et al. [29] could differentiate the aggregation from the hardening stages.

## 5. Conclusions

Coagulation is a critical step for cheesemaking and it cannot be controlled without knowing its mechanisms and the stages that constitute it. However, despite the large number of studies that have been carried out over almost ten decades with the aim of modeling the gel assembly of enzymatically hydrolyzed casein micelles, this process is not yet known or understood in sufficient detail.

On this regard, the main descriptive models for rennet-induced coagulation have been reviewed to evaluate the controversy on the existence of different phases, i.e., κ-casein hydrolysis, destabilized micellar aggregation, and gel hardening. Some authors have recently reviewed the micellar aggregation models, but without differentiating them from hardening. Indeed, most of the proposed models fail to differentiate micellar aggregation and gel hardening as two distinct but, to a certain extent, overlapping processes which are jointly responsible for gel assembly.

A greater research effort is required to obtain models able to reconcile the stages of milk coagulation in a combined model, which integrates the enzymatic hydrolysis of κ-casein and the two stages constituting the non-enzymatic phase of gel formation: aggregation and hardening. Additionally, a successful model should be practical enough for industrial application so that its usage is suitable to anticipate the coagulation progress in the cheese plant for control improvement. Without a theoretical model such as this, the determination of an optimum cutting time during the manufacturing of rennet-induced cheeses will remain empiric.

## Data Availability

No new data were created or analyzed in this study. Data sharing is not applicable to this article.
